# Strained 2D Semiconductor Lateral Heterojunctions via Grayscale Thermal‐Scanning Probe Lithography

**DOI:** 10.1002/smsc.202500404

**Published:** 2026-01-12

**Authors:** Giorgio Zambito, Giulio Ferrando, Matteo Barelli, Michele Ceccardi, Federico Caglieris, Daniele Marrè, Francesco Bisio, Francesco Buatier de Mongeot, Maria Caterina Giordano

**Affiliations:** ^1^ Dipartimento di Fisica Università di Genova Via Dodecaneso 33 16146 Genova Italy; ^2^ CNR‐SPIN Corso Perrone 24 16152 Genova Italy

**Keywords:** 2D transition metal dichalcogenides semiconductors, 3D grayscale nanolithography, few‐layer MoS_2_, Kelvin Probe Force Microscopy, lateral heterojunctions, local strain engineering, thermal‐Scanning Probe Lithography

## Abstract

Nanoscale tailoring of the optoelectronic response of 2D transition metal dichalcogenides semiconductor layers (TMD) is demonstrated thanks to a novel strain engineering approach based on grayscale thermal‐Scanning Probe Lithography (t‐SPL). This method allows the maskless nanofabrication of locally strained 2D MoS_2_‐Au lateral heterojunction nanoarrays that are characterized by lateral modulation of the electronic band structure. 2D MoS_2_ layers are conformally transferred onto grayscale t‐SPL templates characterized by periodic nanoarrays of deterministic faceted nanoridges. This peculiar morphology induces asymmetric and uniaxial strain accumulation in the 2D TMD material, allowing us to tailor their electrical work‐function at the nanoscale level, as demonstrated by Kelvin Probe Force Microscopy. By tailoring the local morphology of the grayscale nanopatterns, the capability to control the strain‐dependent electrical work function of the 2D TMD layers at the local scale is demonstrated. The modulation of the electronic response has been exploited to develop periodic nanoarrays of lateral heterojunctions endowed with asymmetric electrical response by simple maskless deposition of Au nanocontacts onto the strained 2D TMD layers. The locally strained Au‐MoS_2_ layers form asymmetric lateral heterojunctions with strain‐modulated Schottky versus Ohmic behavior, thus representing a promising platform in view of tunable ultrathin nanoelectronics, nanophotonic, and sensing applications.

## Graphical abstract

A novel strain‐engineering approach using grayscale thermal‐Scanning Probe Lithography tailors the optoelectronic response of 2D Transition‐Metal Dichalcogenide (TMD) layers. Nanofaceted templates control local strain in MoS_2_, enabling engineered carrier extraction in asymmetric Au‐MoS_2_ lateral heterojunction nanoarrays, as detected by Kelvin Probe Microscopy. This method offers a promising route for applications in nanoelectronics, nanophotonics, and sensing.

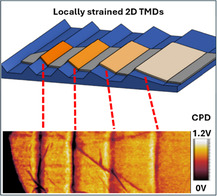

## 1 Introduction

Transition metal dichalcogenides (TMD) recently gathered increasing interest as promising 2D materials for next‐generation ultrathin devices in various fields ranging from optoelectronics and biosensing to quantum technologies.^[^ ^1^, ^2^, ^3^, ^4^, ^5^, ^6^ ^]^ Thanks to their atomic layered structure and tunable electronic band structure, they have been exploited as building blocks for van der Waals heterostructures and represent optimal candidates for ultrathin nanoelectronics and nanophotonics applications.^[^ ^7^, ^8^, ^9^, ^10^, ^11^, ^12^, ^13^, ^14^, ^15^ ^]^ A challenging aspect in this field deals with the fabrication of lateral heterojunctions between 2D materials that show different electronic properties, in order to engineer at will the optoelectronic response of ultra‐thin devices.^[^ ^16^, ^17^, ^18^, ^19^, ^20^, ^21^ ^]^ In parallel, the exceptional mechanical resilience^[^ ^22^, ^23^ ^]^ of 2D materials offers the unique opportunity to tailor their optoelectronic response via strain engineering.^[^ ^24^, ^25^, ^26^, ^27^, ^28^ ^]^ Strain‐dependent optoelectronic and photoemission properties have been observed so far by exploiting different approaches based either on rough and nanopatterned materials,^[^ ^24^, ^29^, ^30^, ^31^ ^]^ or on large‐area bending of the substrate.^[^ ^32^, ^33^, ^34^, ^35^ ^]^ Strain‐induced photonic effects have been recently demonstrated over large areas in self‐organized nanogrooved templates.^[^ ^31^, ^36^, ^37^ ^]^ However, these experiments are typically performed on micro‐ and nanopatterns with poor control on the local features/wrinkles and on the strain‐induced optoelectronic effects. Moreover, from the detection point of view, the optoelectronic properties of strained 2D semiconductors have been generally investigated via Raman and photoluminescence microspectroscopy with diffraction‐limited spatial resolution at the micrometric scale.^[^ ^29^, ^38^, ^39^ ^]^ Few pioneering experiments have been performed at the nanoscale by atomic force microscopy (AFM), probe‐assisted bending with challenging detection that can be achieved either via complex nanomechanical devices or by diffraction‐limited microspectroscopy.^[^ ^23^, ^40^, ^41^ ^]^ Recently few Kelvin‐Probe experiments onto strained 2D TMD layers via micrometric structures^[^ ^42^ ^]^ or via Ion irradiation^[^ ^43^ ^]^ have been performed, showing the potential of this detection approach.

However, the accurate control and tailoring of the intensity and spatial localization of the strain at the nanoscale level are still lacking.

The possibility to reshape fragile 2D layers at the nanoscale in a noninvasive way has been recently achieved by thermal‐Scanning Probe Lithography. This technique allows local and controlled heating of the sample surface with a sharp nanotip and has been employed to perform thermochemical lithography, thermomechanical nanocutting, or straining of fragile 2D layers, showing promising results in photonics.^[^ ^44^, ^45^, ^46^, ^47^, ^48^ ^]^

In this work, we demonstrate the active tailoring of the optoelectronic properties of 2D TMD semiconductor layers at the local scale with the aim of engineering 2D lateral junctions. To achieve this goal, we develop deterministic grayscale periodic templates with faceted morphology by a novel thermal‐Scanning Probe Lithography (t‐SPL) approach, and we employ these platforms for the conformal transfer of 2D MoS_2_ layers. Thanks to t‐SPL, we demonstrate the capability to tailor the faceted morphology, resulting in the control of the local strain‐induced in the 2D material. These strain engineering capabilities have been detected by Kelvin Probe Force Microscopy (KPFM) imaging of the periodically bent 2D layers. This showed asymmetric periodic modulation of the electrical surface potential induced by strain, with spatial resolution at the nanoscale. As a step forward, we exploit such strain‐induced modulation to engineer periodic lateral Au‐MoS_2_ heterojunctions with asymmetric response by simple maskless deposition of Au nanocontact nanoarrays. The KPFM imaging of these ultrathin hybrid platforms demonstrates the periodic sequence of strain‐induced lateral heterojunctions endowed with asymmetric Schottky versus Ohmic response at the alternating Au‐MoS_2_ nanostripe arrays.

## 2 Results and Discussion

**Figure 1:**
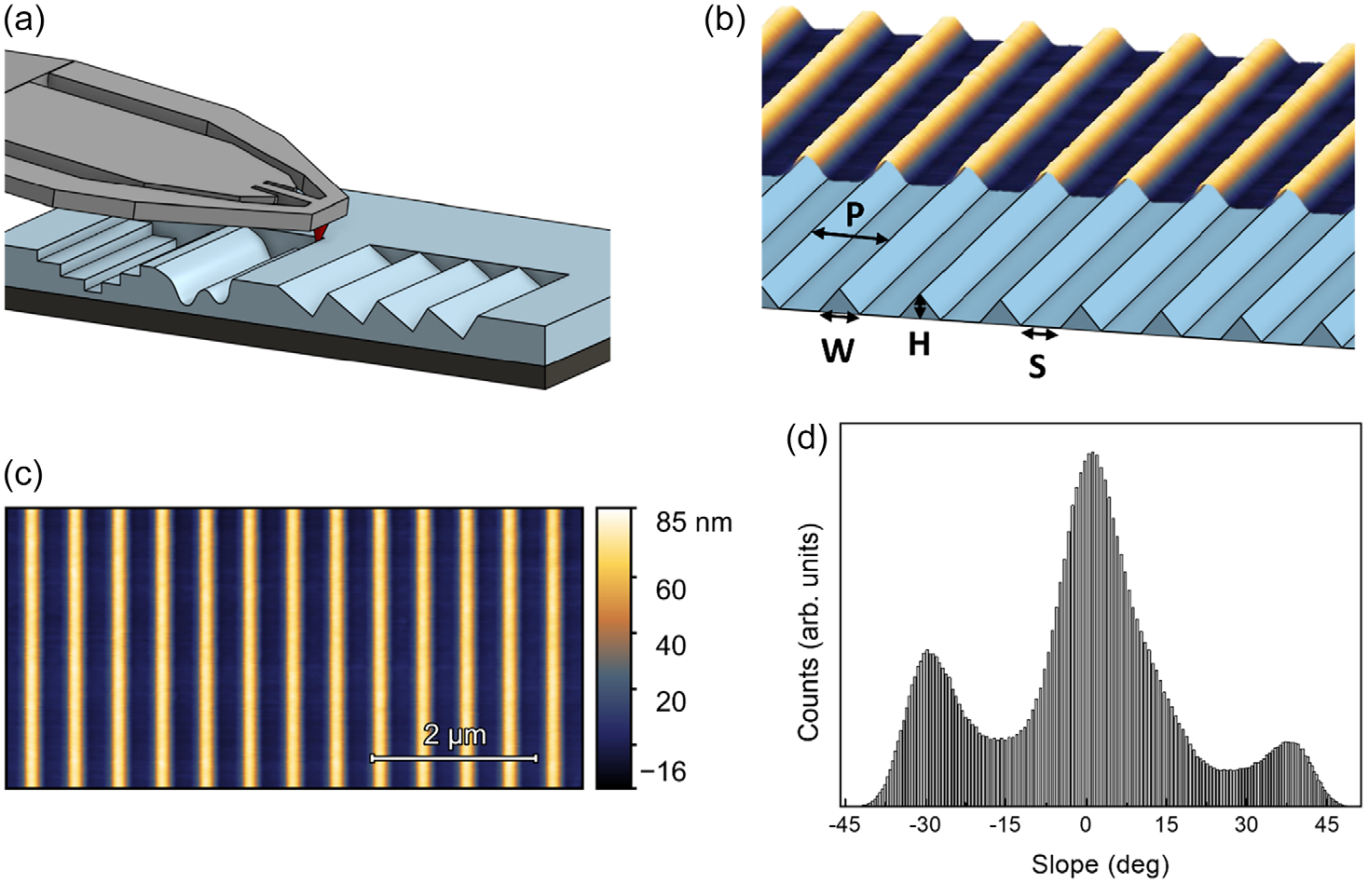
a) Sketch of the grayscale t‐SPL process on a PPA layer. b) 3D view of the faceted nanostructured template of Sample 1 as designed (bottom sketch) and measured by AFM topography after patterning (top). c) Top view of AFM topography after patterning. d) Slope distribution extracted from (c) as the first derivative of the line profiles.

By exploiting the unique capability of the thermal‐Scanning Probe Lithography (t‐SPL^[^ ^49^ ^]^ NanoFrazor instrument) to “write” high‐resolution 3D nanostructures onto thermally labile materials with a hot nanoprobe, we have obtained deterministic grayscale nanotemplates (sketch in **Figure** **1**a) which have been exploited as strain engineering platforms for 2D TMD semiconductors.

To achieve high‐resolution writing performances onto optically transparent substrates, we have exploited glass substrates coated with a thin indium tin oxide (ITO) film, acting as a counter electrode for the nanolithography process, allowing the precise control of the contact force between t‐SPL nanotip and surface.^[^ ^50^ ^]^ We perform t‐SPL on a Polyphthalaldehyde (PPA) polymer layer spin‐coated onto a transparent and conductive ITO thin film supported by a glass substrate (see the Experimental Methods section for details).

We exploited t‐SPL nanolithography to develop grayscale nanotemplates characterized by arbitrarily defined 3D shape, as shown in the topography of Sample 1 (Figure 1b, details about the t‐SPL nanolithography in the Experimental Methods section). Here, the 3D view of the design fed to the NanoFrazor software (light blue sketch) is reported together with the 3D topography image acquired in real‐time by the t‐SPL system during the thermal nanolithography (color map), demonstrating the correspondence between design and patterned nanostructures. As target nanopattern, we chose a faceted nanotemplate that can be exploited to host conformal layered materials, inducing a modulation of their properties thanks to the pattern‐induced strain of the layers. Figure 1c shows the top view of the same AFM image. The highly ordered nanoarrays are characterized by a periodicity of 530 nm and homogeneous height of the nanostructures of 60 nm. We exploit the high control of the t‐SPL tip to fabricate oriented ridges with deterministic tilt angle of the facets, with lateral spatial resolution in the range of 10 nm and with depth‐resolution up to 1 nm.^[^ ^51^ ^]^ The topography image (Figure 1c) highlights the presence of periodically oriented ridges, as demonstrated by the histogram of the slopes (Figure 1d). The latter shows a central maximum at 0° corresponding to the inter‐ridges flat regions, and two further maxima at −29° and +37° corresponding to the right and left nanofacets, respectively. These high‐resolution patterns have been produced over uniform areas extending hundreds of μm^2^ (see Figure S1, Supporting Information), thus showing the stability of the proposed t‐SPL approach for grayscale nanolithography onto transparent dielectric substrates.

**Figure 2:**
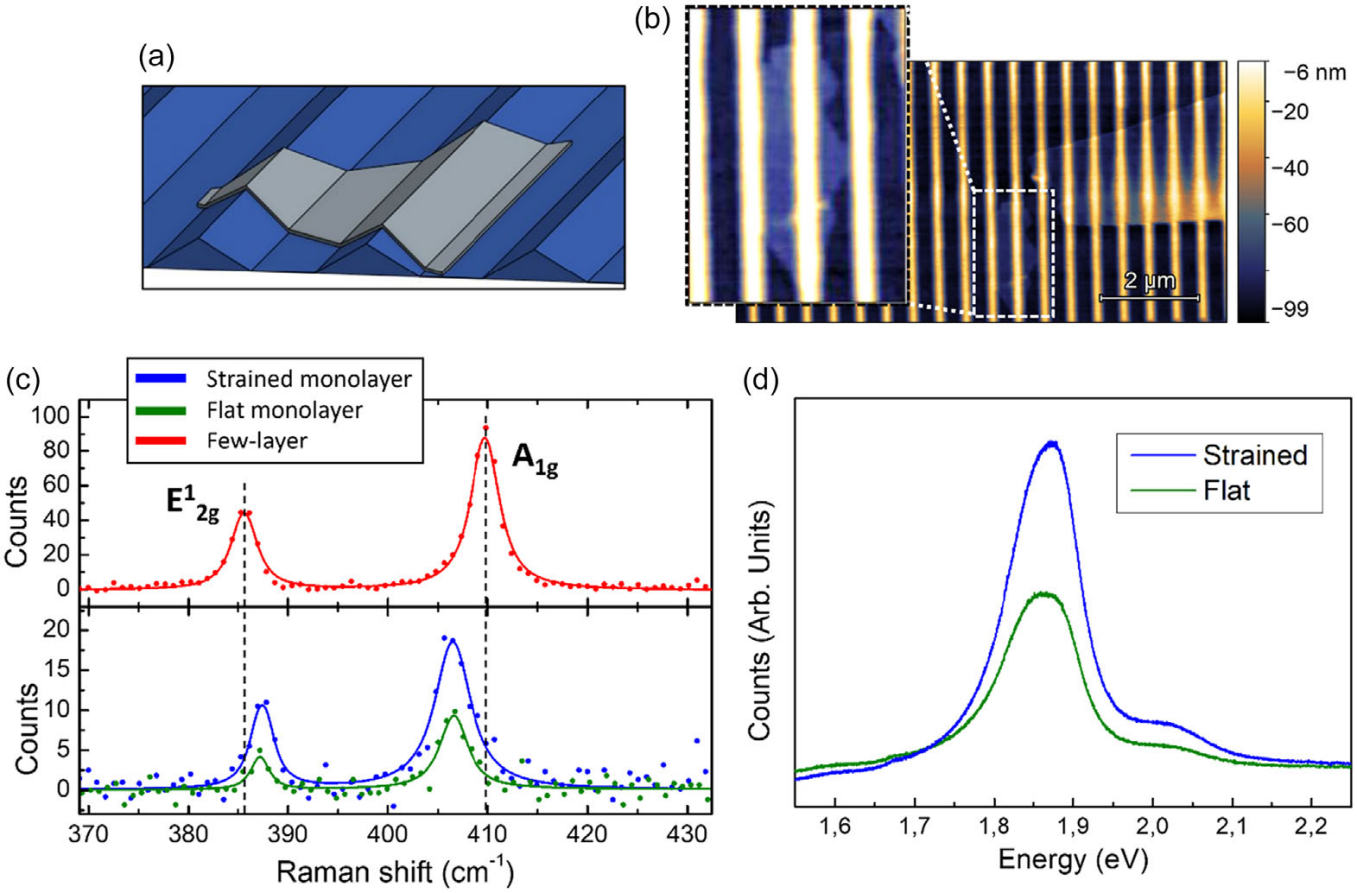
a) Sketch of a 2D MoS_2_ layer conformal to the underlying template. b) AFM image showing few‐layer MoS_2_ flakes conformally transferred onto the grayscale nanotemplate. A zoom on the 2D monolayer is shown in inset with saturated vertical dynamic. c) Raman spectra of the few‐layer MoS_2_ flakes: the strained few‐layer (red line), the strained monolayer (blue line), and a flat monolayer lying on the same substrate (green line) are shown. d) Photoluminescence spectra of the strained (blue line) and flat (green line) MoS_2_ monolayers.

These t‐SPL grayscale templates have been exploited to perform deterministic transfer of mono‐ and few‐layer MoS_2_ semiconductors^[^ ^52^ ^]^ (sketch in **Figure** **2**a), as shown in the AFM image of Figure 2b.

The AFM image shows nanowrinkled few‐layer MoS_2_ flakes transferred onto the 3D faceted t‐SPL template. A 2D monolayer MoS_2_ (highlighted by a white dashed line) and a few‐layer flake are detected, as confirmed by the Raman spectra of Figure 2c. The monolayer conformally follows the faceted periodic topography, as shown in more detail in the zoomed inset image of Figure 2b with saturated vertical dynamic.

In parallel, the wider few‐layer MoS_2_ flake is characterized by a gradually decreasing grade of conformality from top to bottom until it becomes almost completely free‐standing in the lower regions (see AFM line profile in Figure S2, Supporting Information).

The monolayer nature of the flakes is confirmed by micro‐Raman spectroscopy, performed with a Jasco NRS‐4100 confocal Raman spectrometer with a 100× objective (NA = 0.9). All the spectra have been detected by excitation of the 2D layers at 532 nm wavelength (details in the Experimental Methods section). In Figure 2c, the Raman spectra acquired on the few‐layer MoS_2_ flake (red line) and on the strained monolayer MoS_2_ (blue line) are shown together with the spectrum of a flat MoS_2_ monolayer (green line) transferred on a flat area of the same substrate (see image of the flat flake in Supporting Information Figure S3). All the spectra show two maxima that are compatible with the vibrational modes *E* ^1^_2g_ and *A*_1g_ of MoS_2_ layers. The Raman shift of each maximum has been evaluated by a Lorentzian fit of the spectrum, also calculating the spectral shift Δ*K* between the *E* ^1^_2g_ and the *A*_1g_. For the few‐layer flake, the spectral shift reads Δ*K*_few‐layer_ *= *(24.1 ± 0.5) cm^−1^, a value that is compatible with a few‐layer MoS_2_. The spectra acquired on the two monolayers show two maxima at about 387 and 407 cm^−1^, with calculated spectral shifts reading Δ*K*_2D‐strained_ = 19.1 cm^−1^ and Δ*K*_2D‐flat_ = 19.4 cm^−1^ for the strained and flat layer, respectively. These values of Raman shift confirm the monolayer structure of these 2D MoS_2_ flakes.^[^ ^53^ ^]^ Additionally, we observe a factor 2 enhancement of the Raman intensity of the strained monolayer with respect to the flat configuration, in accordance with recent results on strained TMD.^[^ ^54^ ^]^ This first observation suggests a strain‐induced modification of the optoelectronic response of the 2D layer conformal to the nanopattern.

To evaluate the optoelectronic response at the microscale, we detect the photoluminescence (PL) spectra (Figure 2d) acquired both onto the strained and onto the flat 2D monolayer. We detect an enhancement of the photoluminescent emission of the strained 2D layer, with respect to the flat layer, of a factor 2.9 for A‐exciton emission (details in Supporting Information, Figure S4). In both cases, a strong exciton emission is detected at 1.89 eV energy due to the direct radiative recombination of A exciton, superimposed on a contribution of the trion emission at room temperature.^[^ ^55^ ^]^ A weaker emission signal is detected at 2.03 eV, corresponding to the radiative recombination of B‐exciton.

The PL measurements show the capability to modify the photonic response of 2D TMD semiconductor layers by using these periodic faceted nanotemplates. However, to better resolve strain‐induced effects that are expected to occur at the local scale, we investigate the electronic response of the rippled 2D semiconductor layers via KPFM. This scanning probe‐based technique indeed allows the imaging of the surface potential with high spatial resolution.^[^ ^56^ ^]^ We perform KPFM imaging of the wrinkled 2D MoS_2_ in single‐pass mode, which guarantees the colocalized acquisition of topography and surface potential signal by using the Nano‐Observer instrument by CSI (see details of the KPFM in the Experimental Methods section).

**Figure 3:**
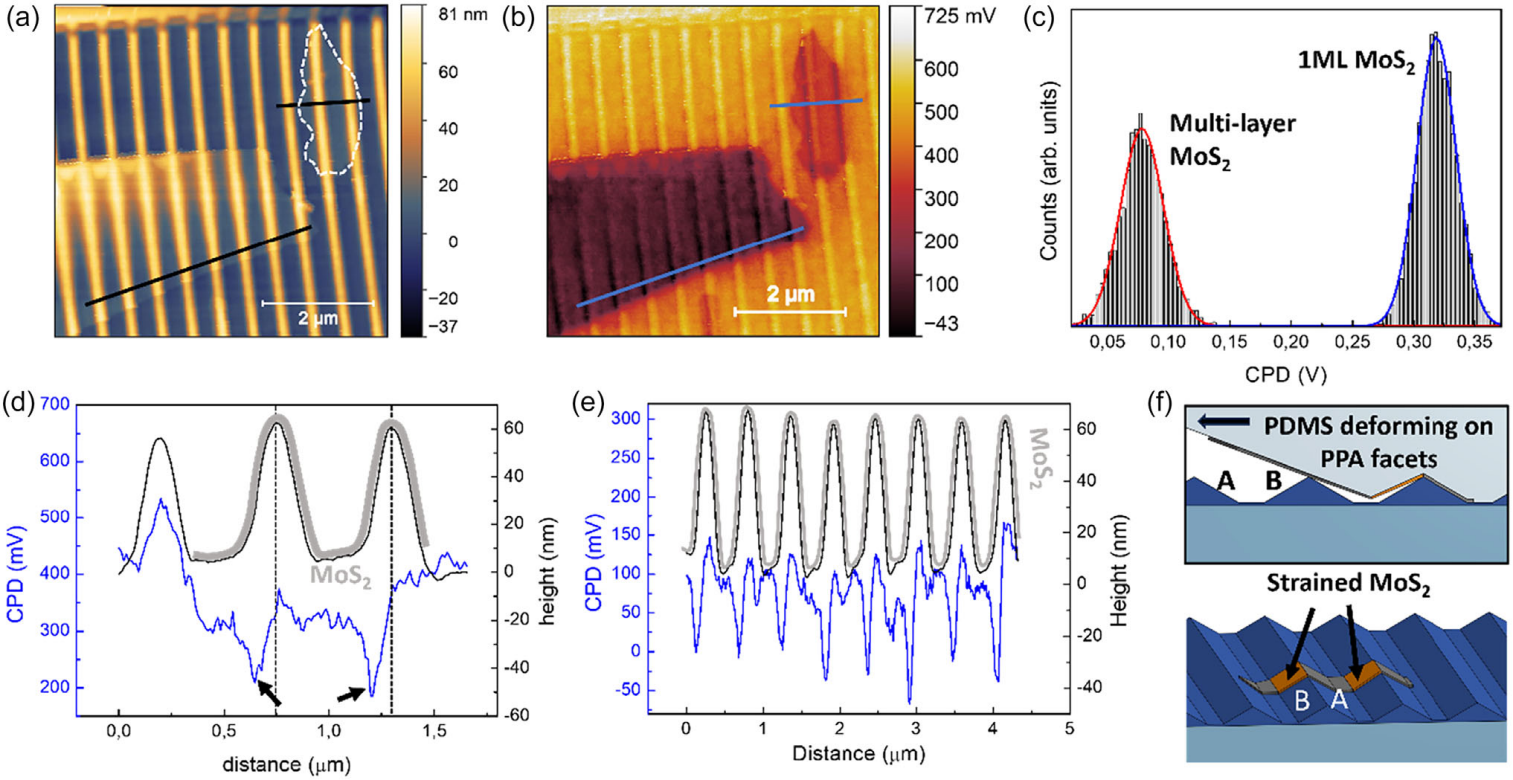
a,b) Colocalized AFM and CPD image of the transferred 2D monolayer and multilayer MoS_2_ flakes detected by single‐pass KPFM. c) Histogram of CPD data extrapolated from selected flat regions of mono‐ and multilayer MoS_2_. d,e) Colocalized topography (black lines) and CPD signal (blue lines) corresponding to mono‐ and multilayer MoS_2_, respectively, extracted from the line profiles highlighted in (a) and (b). The regions where a MoS_2_ layer is present are depicted with thick gray lines. f) Sketch of the strain accumulation effect during PDMS‐mediated transfer of the 2D materials onto the nanotemplate, and periodic modulation of the strain in the nanowrinkled 2D material.

**Figure** **3**a and b show the topography and the colocalized Contact Potential Difference (CPD) in Kelvin Probe Microscopy of the nanowrinkled MoS_2_ layers. The detected CPD signal is defined as follows

(1) $$\text{CPD}=\frac{\phi_{\text{tip}}-\phi_{\text{surf}}}{e}$$

where $\phi_{\text{tip}}$ is the tip work function, and $\phi_{\text{surf}}$ is the work function of the surface, *e* is the elementary charge. A strong CPD contrast of about 100 mV is detected corresponding to MoS_2_ monolayer with respect to the supporting template (Figure 3b), despite its topography contrast being very weak (white dashed line in Figure 3a). In parallel, a strong CPD contrast is also detected corresponding to the few‐layer flake.

The work function difference between monolayer and few‐layer MoS_2_ has been evaluated by considering the flat inter‐ripple regions and extracting the histogram of the detected CPD (Figure 3c), as described in detail in Figure S5 of Supporting Information. A two‐component Gaussian fit is calculated in correspondence to the lower and a higher CPD distributions that correspond to the few‐layer (red line) and to the monolayer MoS_2_ (blue line), respectively. The gap between the two distributions reads Δ_CPD_ = 240 mV, which corresponds to the work function difference between flat monolayer and few layer MoS_2_.^[^ ^57^ ^]^ Remarkably, in the CPD image of Figure 3b, the signal of both the monolayer and the few‐layer MoS_2_ also shows a periodical modulation in register with the underlying nanopattern. Asymmetric CPD dips are detected on the left facet of each nanoridge, as shown by the line‐profiles of Figure 3d and e (blue lines), respectively corresponding to the strained monolayer and few‐layer MoS_2_. The periodic surface potential dips read about 130 mV for monolayer, and 150 mV for few‐layer MoS_2_, and are detected with high spatial resolution at the level of few tens of nanometers. These characteristic periodic features are due to the conformal adhesion of the 2D layer onto the template and homogeneously run along the whole rippled 2D layer structure in register with the pattern (darker regions in Figure 2b, further CPD line profiles in Figure S6 of Supporting Information). Indeed, for few‐layer MoS_2_, the modulation of the CPD is reduced near the edges of the flake, where conformality is not fully preserved, as shown in Figure S7, Supporting Information.

This periodic and asymmetric modulation of the surface potential can be attributed to template‐driven strain, rising during transfer of the 2D TMD layers from the soft polydimethylsiloxane (PDMS) elastomeric stamp. The release of the 2D materials onto the periodic templates occurs along a direction perpendicular to the templates ridges, thus inducing an asymmetric field strain in the material, as shown by the sketches in Figure 3f. Indeed, the employed setup allows the control of the PDMS wavefront propagation during transfer, ensuring the latter to take place perpendicularly to the pattern ridges (more details in Figure S11, Supporting Information). Periodic CPD minima shown in Figure 3d and e can be attributed to a pattern‐modulated tensile strain localized along the left side of each nanoridge. The electrical work function increase (i.e., CPD decrease) detected along the left side nanofacets can thus be associated with a tensile strain accumulation, as also shown in recent simulation studies.^[^ ^25^ ^]^

In order to control the strain of 2D TMD layers at the local scale, and to further confirm the detected effect, we engineer a new template (Sample 2) with increased height and tailored local tilt and inter‐ridges spacing (details about this nanofabrication experiment in Experimental Methods).

**Figure 4:**
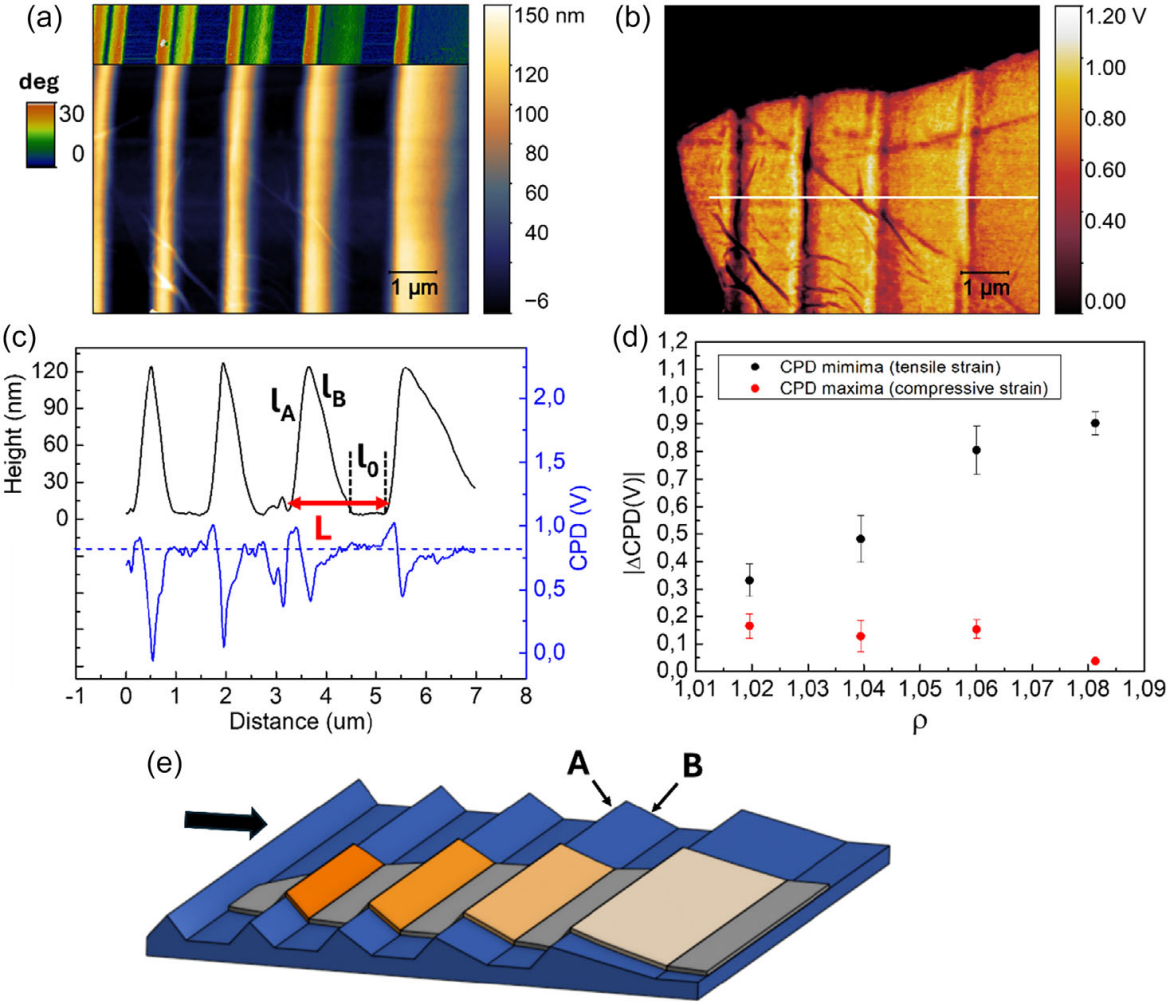
a,b) Colocalized AFM image and CPD map of a new t‐SPL faceted template (Sample 2) hosting a conformal 2D TMD layer. A portion of the local slope map is shown in inset of panel (a). c) Colocalized topographic cross‐section profile (black line) and CPD signal (blue line) extracted from images a,b). d) Graph showing CPD variations with respect to the flat condition (absolute values) as a function of the ridge geometrical ratio *ρ* (mean ± SD, *n* = 4). Black data points correspond to CPD minima (tensile strain, associated with the variable‐tilt B facets), while red data points correspond to CPD maxima (compressive strain, associated with the constant‐tilt A facets). e) Sketch of the strain accumulation effect during PDMS‐mediated transfer of the 2D materials onto the nanotemplate, and periodic modulation of the strain in the nanowrinkled 2D material. Black arrow indicates the transfer direction of the flake on top of the pattern.

The topography of this t‐SPL nanopattern hosting a conformal MoS_2_ layer is shown in **Figure** **4**a, together with a portion of the corresponding map of the local slope. The template is characterized by engineered tilt of the right‐side facets of the nanoridges, tuned from 25° to 5° in 5° steps, while the tilt of the left‐side facets is kept constant at 25°. In particular, the first ripple on the left side of the image is characterized by a symmetric profile with tilt of both its ridges of 25°, while the following nanostructures are characterized by asymmetric profile with local slope of their right‐ended facets that gradually decreases. This peculiar modulation is transferred to the 2D TMD flakes that conformally follow the underlying topography. The 2D TMD layer (colored regions in Figure 2b) is clearly detected in the colocalized CPD map, with strong contrast with respect to the bare PPA template regions (black regions). Additionally, a periodic modulation of the CPD signal is detected onto the 2D TMD in registry with the topographic pattern, as also confirmed by the colocalized topographic and CPD profiles of Figure 4c (black and blue line, respectively). A strong CPD asymmetric minimum, whose intensity reaches 0.8 V, is detected near the ridges apex with a long tail spreading along the right‐side facets. This CPD decrease can be associated with a tensile strain accumulation in these regions. In parallel, a weaker CPD maximum of about 0.1/0.2 V is measured, corresponding to the left‐side facets that can be explained in terms of a weaker compressive strain accumulation effect. We stress that the results of this last experiment are coherent with those shown in Figure 3 since the flake has been transferred following opposite directions in the two cases, as shown by the arrow in Figure 3f and 4e, respectively. The CPD profiles coherently show a weak compressive strain localization along the facets that are directly oriented toward the transfer direction (facets A), while a strong tensile strain localization is detected along the opposite ridges (facets B). Additionally, in the second experiment, we show the capability to tune the amplitude and the spatial extension of the strain‐induced CPD signal by simply tailoring the tilt angle of the nanoridges. Indeed, CPD map and line profile of Figure 4b,c show that by simply changing the tilt of the TMD strained facets at fixed height of the pattern, a decrease of the CPD minimum with a broadening of the feature along the whole ridge is detected. This is an indication of the crucial role played by the underlying pattern in the complex interplay between layer adhesion to the template and slipping effects of the 2D TMD along the nanogrooved profile.^[^ ^58^ ^]^

In our experiment, the difference between the elastic properties of the PDMS stamp supporting the flat 2D TMD and the PPA template allows the conformal transfer of the 2D layers onto the faceted nanopatterns. The Young modulus of the PDMS and of the PPA reads about 8 10^−4^ and 0.3 GPa, respectively,^[^ ^59^, ^60^ ^]^ thus suggesting that the PDMS stamp strongly deforms onto the nanostructured PPA template characterized by higher stiffness. As shown in the sketches of Figure 3f and 4e, the 2D layer first lies onto the facets tilted toward the transfer direction (A facets) with small compressive deformation, then it follows the PDMS conformal stretching onto the opposite ridges (B facets) of the nanotemplate. After the complete conformal adhesion of the flake, the PDMS is released, leaving a nonhomogeneous tensile strain in the 2D layer that is localized along the B facets and follows the periodic modulation of the underlying nanopattern. This strain field is highly localized in the case of steep short facets, while its spatial distribution becomes broader along the wider facets that emerge as their tilt angle decreases.

As a first estimate, the degree of strain of the 2D TMD can be evaluated by considering the geometrical ratio $\rho =\frac{l_{A}+l_{B}+l_{0}}{L}$, where *l*_A_ *, l*_B_, and *l*_0_ are the width of facet A, facet B, and of the flat inter‐facet regions, while *L* is the peak‐to‐peak distance, as highlighted in Figure 4c. We stress that the geometrical ratio *ρ* represents an upper limit of the strain in case the TMD film is pinned at the top ridges, while slippage between TMD and PPA during the transfer process reduces the effective strain accumulation into the semiconductor layer. The plot of the absolute value of the CPD difference, |ΔCPD|, corresponding to the CPD minima (tensile strain, black dots), and to the CPD maxima (compressive strain, red dots), is shown in Figure 4d. This plot has been extracted considering different CPD profiles and calculating the mean values of each target feature. ΔCPD corresponding to the maxima (compressive strain) is characterized by a constant behavior, while an increase of ΔCPD corresponding to the minima (tensile strain) from 0.3 V to about 0.9 V is detected as the geometrical ratio ρ increases from 1.02 to 1.08.

These results demonstrate the capability to tailor the strain in 2D TMD layers by exploiting engineered grayscale nanotemplates, thus obtaining a controlled tuning of their electronic response, locally probed by Kelvin Probe Microscopy.

**Figure 5:**
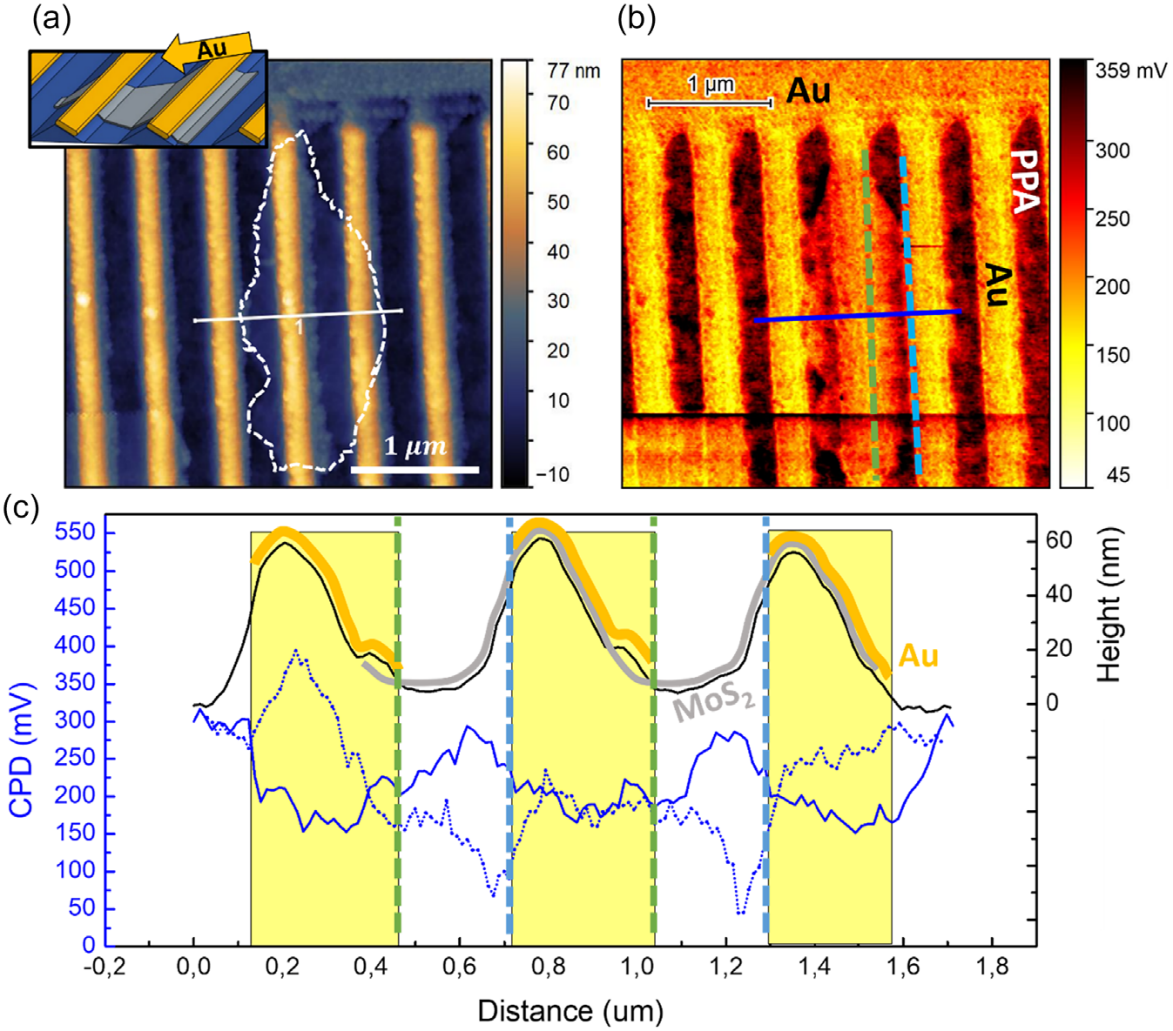
a,b) Topography, sketch, and CPD map of hybrid Au‐MoS_2_ nanoarrays. c) Colocalized line profiles extracted from maps in (a) and (b). Dashed and solid blue lines represent CPD profiles measured before and after Au nanowire deposition, respectively. Regions corresponding to MoS_2_ and Au‐MoS_2_ nanosystems are highlighted with gray and yellow lines, while green and light blue dashed lines in (b), (c) correspond to left and right Au‐MoS_2_ lateral heterojunction.

The peculiar, tilted configuration of these 2D TMD layers offers the possibility to engineer hybrid 2D‐metallic nanoarrays within a simple maskless metal deposition. Under this condition laterally disconnected Au nanowires (NWs) arrays  that can act as local contact for specific portions of the 2D TMD layers characterized by different degree of strain, have been engineered with a maskless approach. Glancing angle Au evaporation perpendicular to the ripple ridges direction, at 80° with respect to the surface normal (sketch in inset of **Figure** **5**a), have been performed on Sample 1, obtaining periodic nanoarrays based on hybrid 2D‐metallic heterostructures. The effective confinement of gold NWs is confirmed by high‐resolution AFM imaging and SEM characterization shown in Supporting Information, Figure S9. The conformal adhesion of the 2D TMD layers onto the template allows us to decorate the 2D TMD semiconductor layers with the periodic Au NWs that act as local nanocontacts for the 2D material, as shown by the AFM topography of Figure 5a. This approach brings a twofold advantage since i) the Au NWs acting as conducting nanoelectrodes are deposited directly on the 2D layers without need for further lithographic steps, thus avoiding unwanted doping and/or damaging of the fragile 2D material. Additionally, ii) the NWs electrodes address portions of the 2D material which are locally strained and show template‐modulated electrical work function.

To investigate the electrical behavior of this novel configuration based on hybrid 2D TMD‐metallic layers, we perform local KPFM measurements. The KPFM map of Figure 5b shows a strong CPD contrast with periodic behavior colocalized with the topography image of Figure 5a. Tilted Au nanostripes (bright regions) are detected with high spatial resolution in both the topography and in the CPD map (Figure 5b). These NWs are characterized by a typical width of about 300 nm and are decorated at their edge by chains of Au nanoclusters that are resolved in both the topography and the CPD map (inverted color scale). The CPD is lower on the Au NWs and reads about 170 mV, while it reads about 315 mV on the bare PPA template. The hybrid 2D‐metallic layer is detected at the center of the image (dashed white line in the topography), inducing further modulation of the detected CPD signal. This way, a novel configuration based on electrically asymmetric Au‐MoS_2_ lateral heterojunctions nanoarrays is achieved. The colocalized CPD map (Figure 5b) and a specific line profile (Figure 5c) show a periodic modulation of the electric response at the nanoscale, in register with the template. A lower CPD value, reading about 200 mV, is detected at the left Au‐MoS_2_ heterojunction (green dashed lines in Figure 5b and c), while a higher CPD value reading about 290 mV is detected at the right MoS_2_‐Au heterojunction. This asymmetric electrical behavior is homogeneously measured onto the whole 2D‐metallic hybrid structure, confirming the capability to tailor at will the electronic properties by the devised strain engineering approach.

To understand this effect in Figure 5c, we compare the CPD profile of the Au‐MoS_2_ heterojunctions (continuous blue line) with the corresponding CPD profile detected on the bare strained MoS_2_ (dashed blue line extracted from Figure 3d). The two detected CPD profiles have been normalized to the work function of the tip ($\Phi_{\text{tip}}$) corresponding to the CPD map of Figure 5b (details in Supporting Information Figure S10). The value of MoS_2_ surface potential at the left heterojunction (dashed green lines in Figure 5c) does not change due to Au NWs deposition, reading a constant value of about 200 mV. This suggests the presence of an Ohmic junction given by the local match of the electronic work functions at the Au‐MoS_2_ lateral interface. At the right heterojunction (dashed blue line in Figure 5c), we observe a strong increase of the surface potential that can be attributed to a built‐in Schottky barrier at the strained MoS_2_‐Au lateral interface.^[^ ^61^ ^]^ This effect can be explained in terms of the strain‐induced modulation of the Fermi Level Pinning and of the Charge Neutrality Level, as recently reported.^[^ ^62^, ^63^ ^]^

This proof‐of‐concept platform based on hybrid 2D‐metallic heterostructures has shown a promising electronic response characterized by periodic Ohmic versus Schottky electrical contact. This effect has been investigated from the fundamental point of view by exploiting a maskless and noninvasive nanofabrication method based on the glancing angle deposition. Future efforts in view of the fabrication of optoelectronic devices should aim at the interconnection of the hybrid TMD‐metal nanocontacts with external circuit pads.

## 3 Conclusion

The controlled strain engineering of 2D TMD semiconductor layers has been demonstrated by exploiting a novel approach based on the grayscale t‐SPL. Under this condition, we show the possibility to tune the electronic properties of 2D TMD at the local scale by simply tailoring the shape of faceted t‐SPL nanotemplates that host conformal MoS_2_ layers.

Periodic grayscale nanotemplates are fabricated by t‐SPL, achieving deterministic faceted nanoridges with nanoscale lateral and vertical spatial resolution. These peculiar 3D templates are exploited for the transfer of 2D MoS_2_ semiconductor layers that conformally follow the underlying faceted templates. These way, the 2D TMD monolayer shows an amplification of the photoluminescence signal detected at the microscopic scale, suggesting strain accumulation in the 2D material. To locally resolve this effect, we perform KPFM imaging of the surface, detecting a periodic modulation of the electrical work function which is asymmetric with respect to each faceted nanoridge and depends on the direction of transfer of the 2D TMD layers. This pattern‐induced effect, attributed to the strain localization, has been further investigated by tailoring the morphology of the faceted nanotemplates. Under this condition, we show the capability to tailor the strain at the local scale, detecting a pattern‐driven modulation of the electrical work function of the material, $\phi_{\text{2D TMD}}$. In particular, a relatively small decrease of $\phi_{\text{2D TMD}}$ (CPD maxima), due to a compressive strain, has been detected along the facets A that are directly oriented toward the direction of transfer, while a stronger increase of $\phi_{\text{2D TMD}}$ (CPD minima), due to a tensile strain localization, has been measured along the opposite facets B. Remarkably, along these latter facets, we show the controlled tuning of the electrical work function by simply tailoring their local geometry, which directly affects the tensile strain localization in the 2D TMD.

Finally, this local modulation of the electronic properties, induced by the faceted templates, allowed us to develop lateral heterojunction nanoarrays based on hybrid Au‐MoS_2_ layers. Thanks to the faceted morphology, these hybrid nanoarrays have been achieved by non‐invasive maskless glancing angle metal deposition onto the template without affecting the quality of the 2D material. This platform shows periodic lateral Au‐MoS_2_ heterojunctions driven by the template, characterized by asymmetric Ohmic versus Schottky electronic behavior, thus demonstrating the possibility to optimize the carrier extraction properties in a proof‐of‐concept configuration.

These promising results thus highlight the potential of the proposed t‐SPL‐based strain engineering approach in view of novel ultra‐thin optoelectronic devices, with impact in many fields ranging from nanoelectronics and nanophotonics to energy conversion and sensing.

## 4 Experimental Section

4.1

4.1.1

##### Sample Preparation and t‐SPL Nanofabrication

t‐SPL has been performed by using NanoFrazor Scholar instrument from Heidelberg Instruments Nano. This technique exploits a Si microchip terminating with a sharp conductive tip that allows for the achievement of local sublimation of thermally labile polymer films. Indeed, the Nanofrazor instrument can provide controlled heat stimulus to the tip that reaches temperature in the range of 800–1100 °C. Under this condition, a feedback‐based nanowriting process can be performed with high efficiency when the substrate is electrically conductive, thus allowing the active control of the tip‐sample interaction. In our experiment, the conductive sample‐holder of the NanoFrazor, acting as counter‐electrode for the nanotip, has been electrically connected to the ITO‐coated substrates. Under this condition, both the lateral and the vertical dynamic of the target nanopattern can be controlled at the local scale, thus allowing us to achieve high‐resolution grayscale nanolithography.

Glass substrates (15 × 20 mm^2^ from Ossila) coated with 100 nm thick ITO film have been exploited for sample fabrication. Since grayscale lithography by t‐SPL requires a conductive counter‐electrode, a silver paste electrode deposited onto the conductive film has been exploited.

A PPA film of 160 nm (sample 1 shown in Figure 1, 2, 3, 4, 5) and 250 nm (sample 2 shown in Figure 4) thickness is deposited on the sample by spin coating of an 8% PPA:Anisole solution prepared with Phoenix 81 powder by AllResist. Spin coating is followed by annealing at 110 °C. Reported thickness values are observed by AFM imaging of the film across a scratch on the film, exposing the underlying substrate with a sharp edge.

3D modeling of the faceted templates has been performed using Onshape software and later converted into grayscale images for t‐SPL use.

##### Exfoliation and Deterministic Transfer of 2D TMD Layers onto T‐SPL Nanopatterns

Exfoliation of 2D MoS_2_ layers was performed by both scotch tape (Nitto SPV224 PVC tape) and PDMS from a bulk MoS_2_ crystal (commercial material by SPI). The scotch tape was used for the initial reduction of the thickness of macroscopic single crystals down to sub‐micrometric range. Then, the PDMS was exploited to obtain atomically thin flakes. In a final step, the 2D MoS_2_ layers have been transferred onto the t‐SPL nanopattern using a deterministic viscoelastic transfer set‐up (Figure S11, Supporting Information).

##### Kelvin Probe and Atomic Force Microscopy (AFM) Characterization

KPFM analysis is performed in ambient conditions by using CS Instruments (CSI) Nano Observer II instrument, operating in KPFM‐Single Pass mode. Electrical contact is formed between the grounded sample holder and the ITO surface by use of a copper wire and silver paste. For the characterization, we used Pt‐coated AFM tips (ANSCM‐PT) from AppNano.

AFM characterization of the sample has been performed by Neaspec instrument (Attocube).

##### Statistical Analysis

Data analysis of AFM and KPFM data has been performed via Gwyddion open‐source software, providing histogram of the local slopes of the detected topographic images and of CPD data, extraction of topography and CPD line profiles, and optimization of vertical dynamics of AFM and KPFM images.

Data analysis and fitting have been performed using OriginPro and Spectra Manager softwares.

## Supporting Information

Supporting Information is available from the Wiley Online Library or from the author.

## Conflict of Interest

The authors declare no conflict of interest.

## Supporting information

Supplementary Material

## Data Availability

The data that support the findings of this study are available from the corresponding author upon reasonable request.
