# Supplementary material for: Strained 2D Semiconductor Lateral Heterojunctions via Grayscale Thermal‐Scanning Probe Lithography
Source: Small Sci. 2026 Jan 12;6(2):e202500404. doi: 10.1002/smsc.202500404 (PMC12915091; doi:10.1002/smsc.202500404)
Supplement: Supplementary file 1 — Supplementary Material [file SMSC-6-e202500404-s001.pdf]

Supporting Information

Strained 2D semiconductor lateral heterojunctions via grayscale thermal-Scanning Probe Lithography

Giorgio Zambito[1], Giulio Ferrando[1], Matteo Barelli[1], Michele Ceccardi[1], Federico Caglieris[2], Daniele Marre[1], Francesco Bisio[2], Francesco Buatier de Mongeot[1], Maria Caterina Giordano[1]*

Supporting Information

Figure S1: Template nanopatterning on areas exceeding hundreds of μm^2:

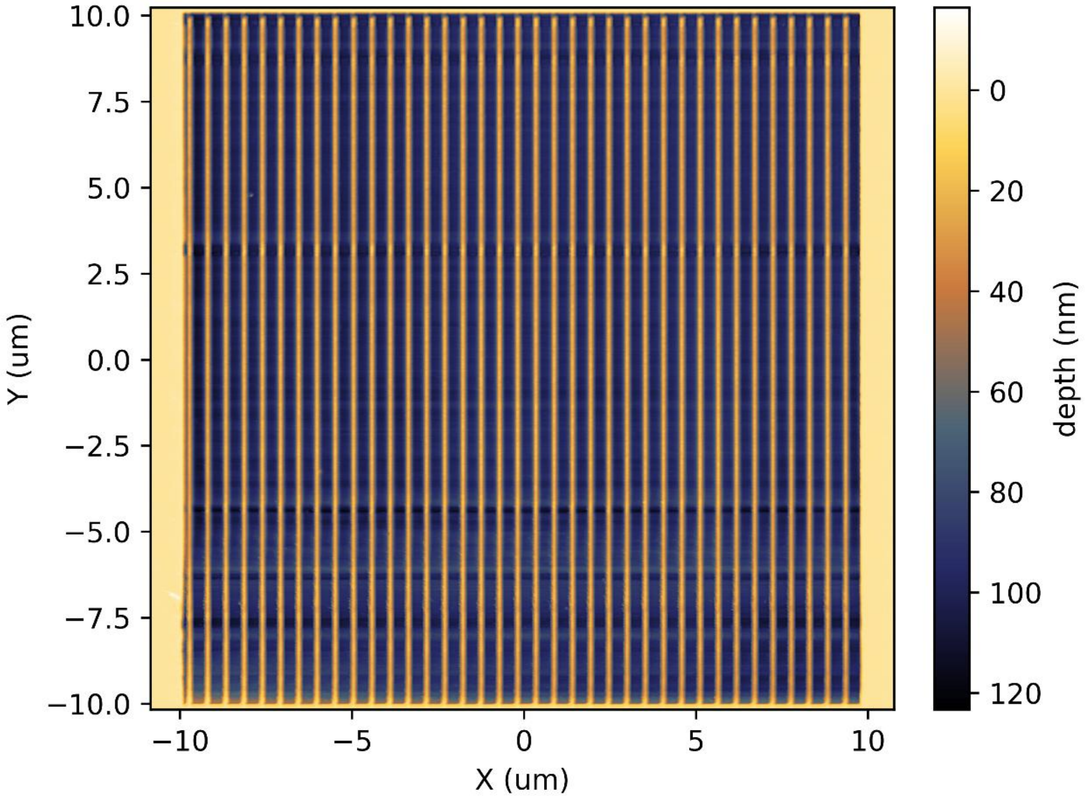

Figure S1: AFM topography of one of the employed nanotemplates, showing the stability of the patterning technique on an area of around 400 μm^2

Figure S2: Conformality of MoS_2 flakes to the nanotemplate

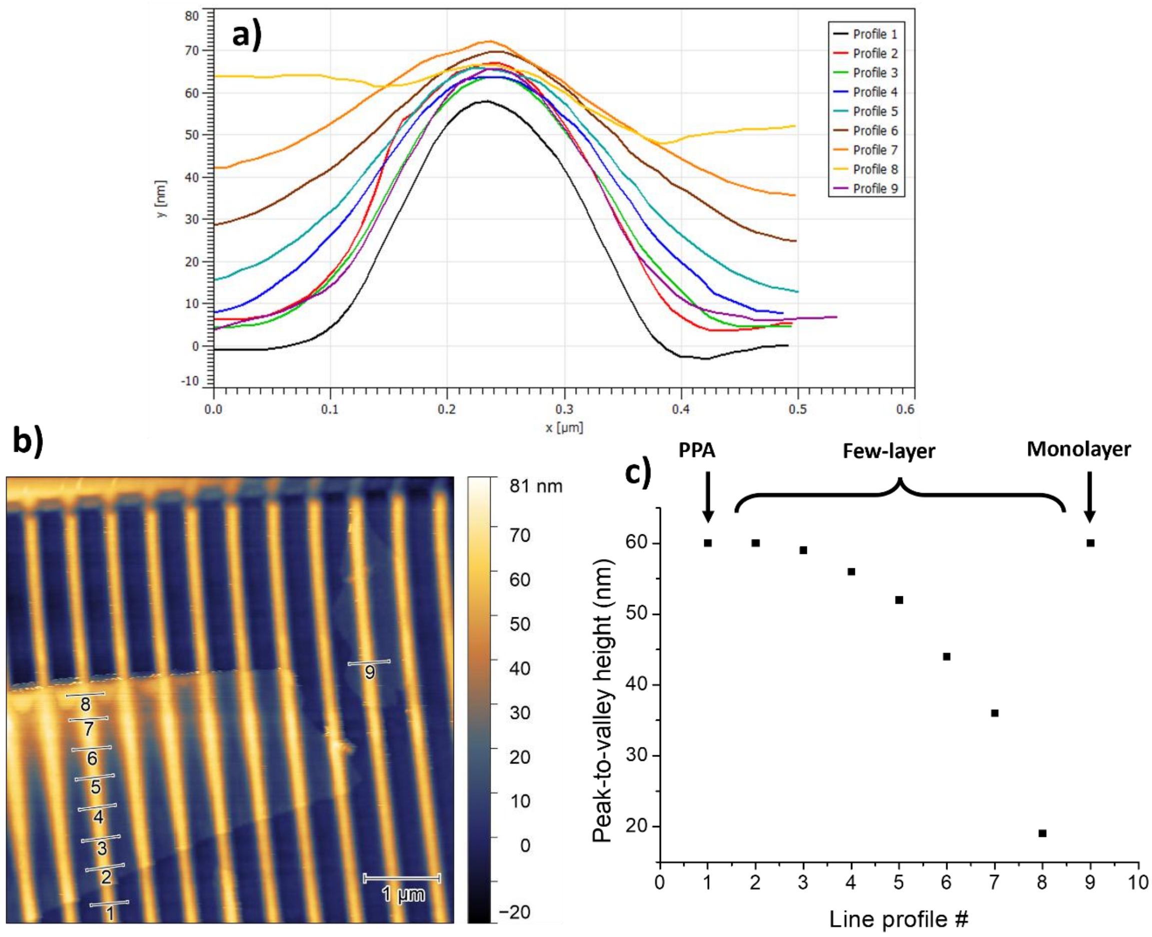

Figure S2: a) Topography line profiles extracted from the AFM image of Figure 3a in the manuscript. Locations of the extracted line profiles respect to the topography AFM map are reported in b). c) Peak to valley heights as measured from line profiles in a).

Figure S3: Flat MoS_2 monolayer on unpatterned PPA

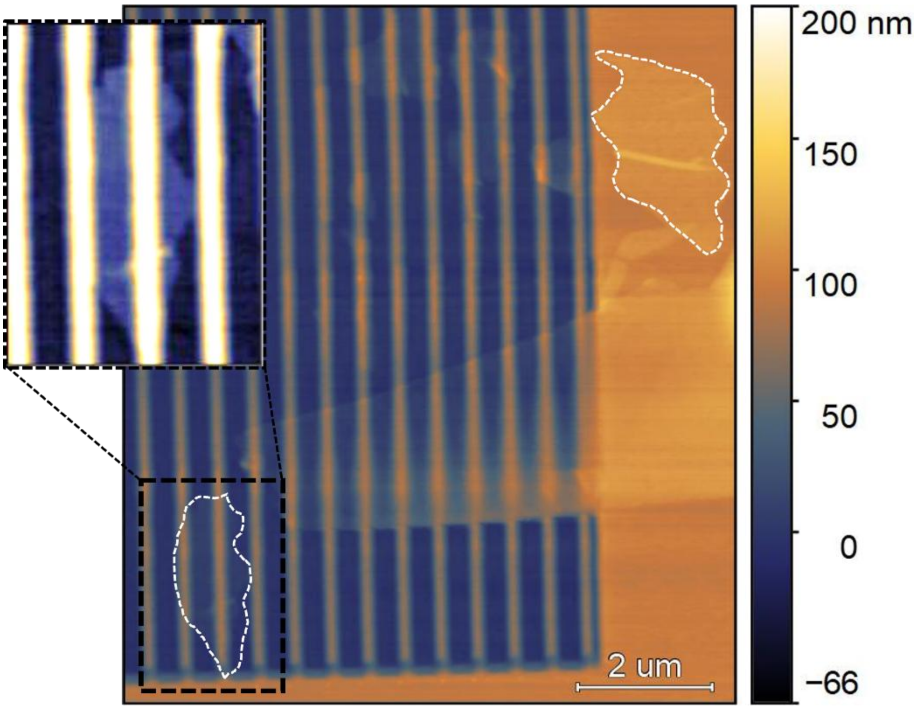

Figure S3: Extended view of the AFM map reported in Figure 2b in the manuscript, showing the presence of a transferred flat monolayer on unpatterned PPA. Raman and PL spectra of this flake are reported in Figure 2c-d in the manuscript.

Figure S4: Fit of PL spectra

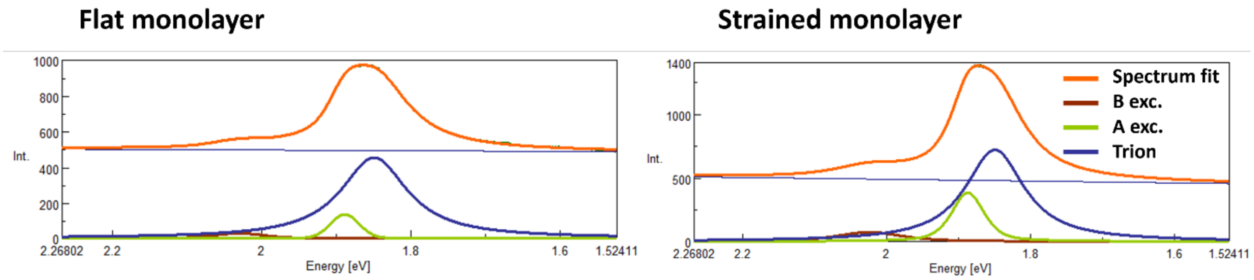

Figure S4: Fitting results of the PL spectra shown in Figure 2d. The orange line represents the total spectrum fit, while the brown, green, and blue lines correspond to the three Gaussian-Lorentzian components for the A and B excitons and the trion emission. For the two spectra, acquired under identical experimental conditions, the A-exciton emission peak reaches heights of 133 and 380 counts for the flat and strained conditions, respectively, resulting in an enhancement factor of 2.9.

Figure S5: Histogram of CPD values for flat mono- and few- layer MoS_2

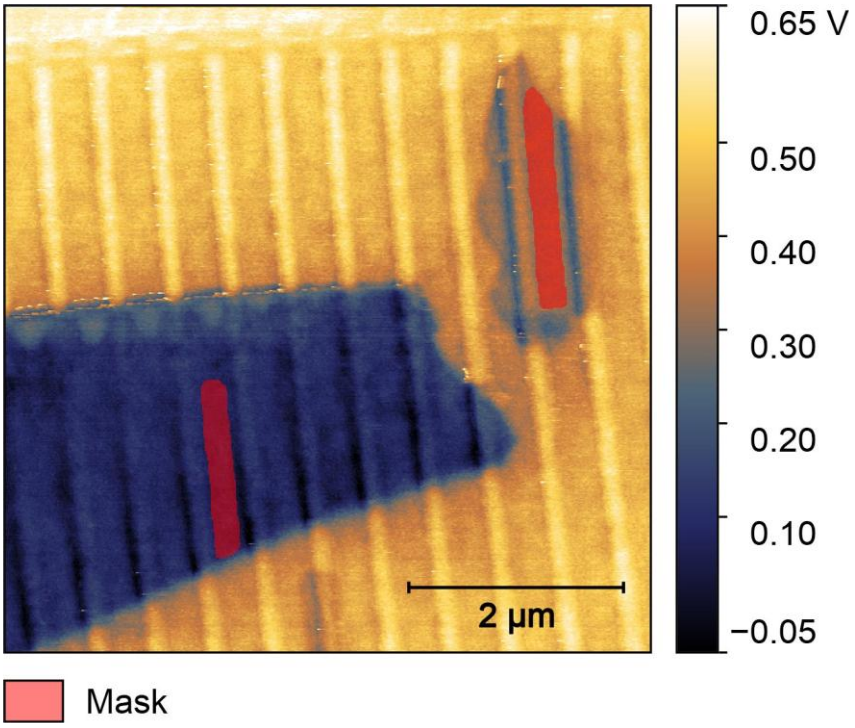

Figure S5: This CPD map (already shown in Figure 3b in the manuscript with different color scale) shows with a red overlayed mask the portions considered to plot the CPD histogram reported in Figure 3c. Only flat portions of both flakes have been selected to gather CPD informations on flat mono- and few-layer MoS2 in the same KPFM map.

Figure S6: CPD profiles

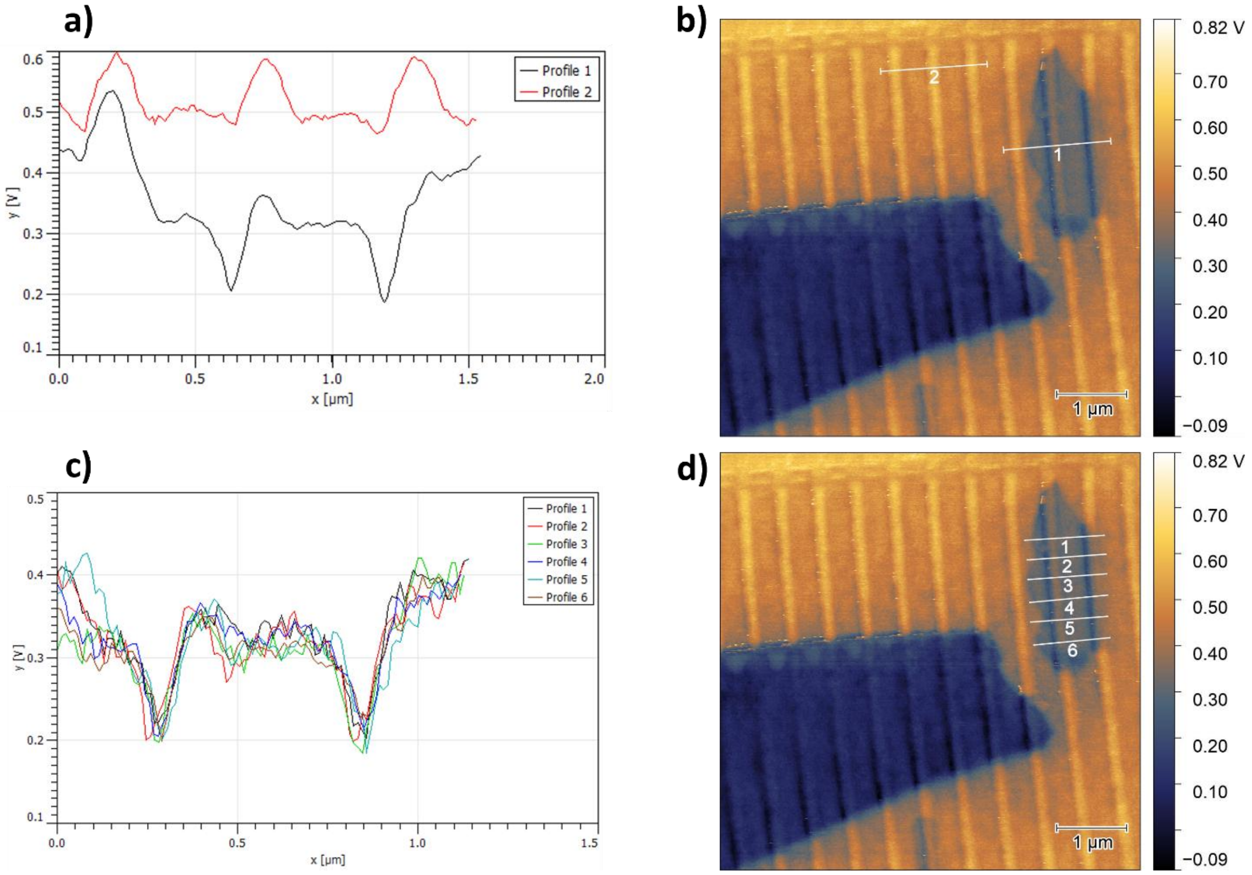

Figure S6: a-b) CPD profiles extracted from bare patterned PPA and strained conformal monolayer, showing the difference in the measured surface potential. The CPD asymmetry respect to the single ridges is only visible on the strained MoS_2 and absent on bare patterned PPA. c-d) Multiple profiles extracted from the strained monolayer, showing that the CPD periodic features discussed in the manuscript run along the whole rippled 2D layer structure.

Figure S7: CPD modulation on regions with different degrees of conformality

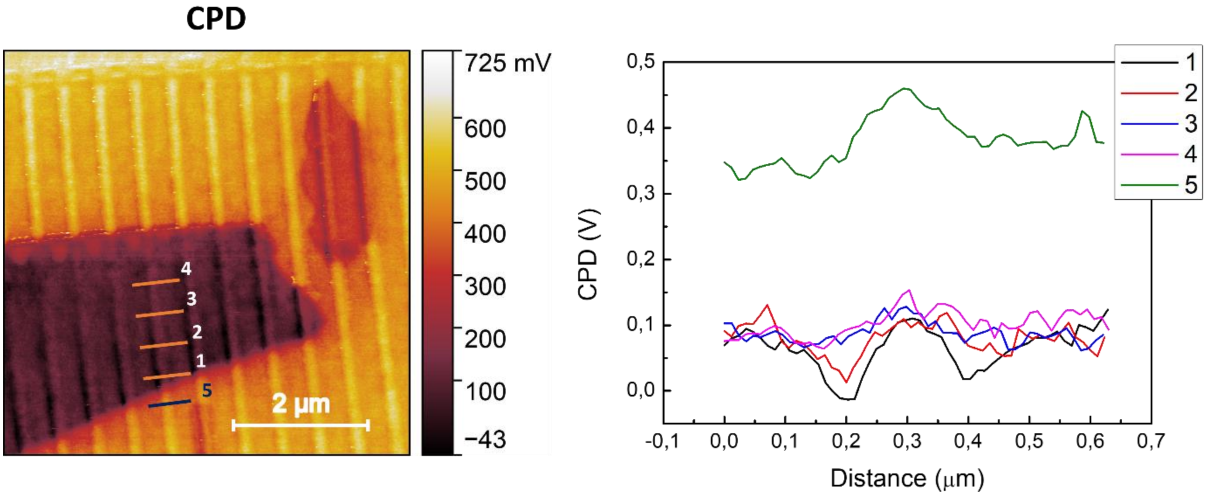

Figure S7: a-b) CPD map and corresponding CPD line profiles extracted from regions of multi-layer MoS_2 with different degree of conformality. A strong CPD modulation is detected in conformal regions (profiles 1, 2) while weaker features are detected in the other cases.

Figure S8: Direction of transfer respect to pattern ridges

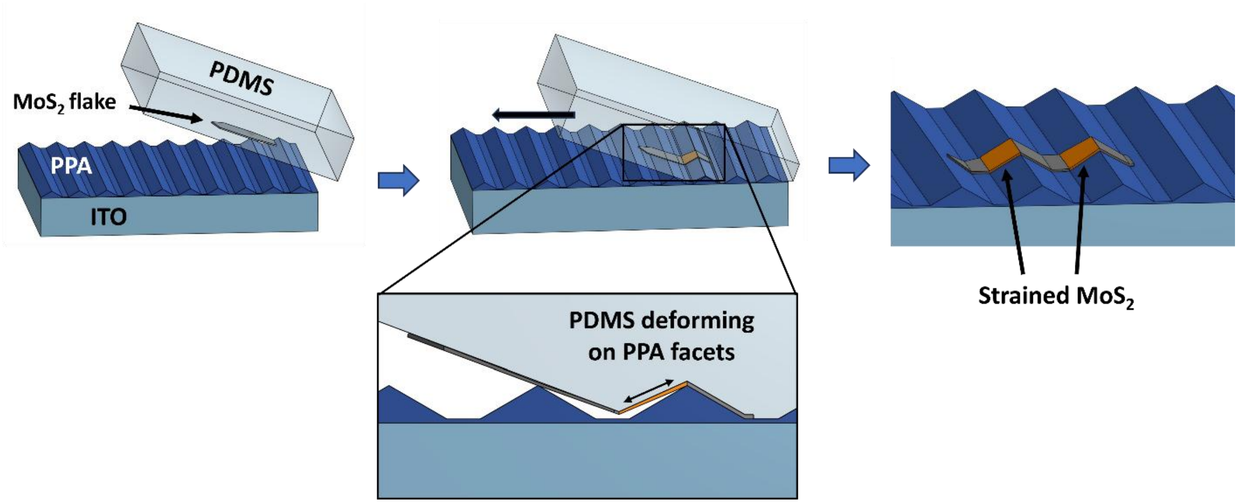

Figure S8: Schematic view of the directional transfer of MoS2 flakes along the faceted patterns, showing the mechanism behind asymmetric localized straining of the MoS2 on the left facets

Figure S9: Characterization of Au Nanowires

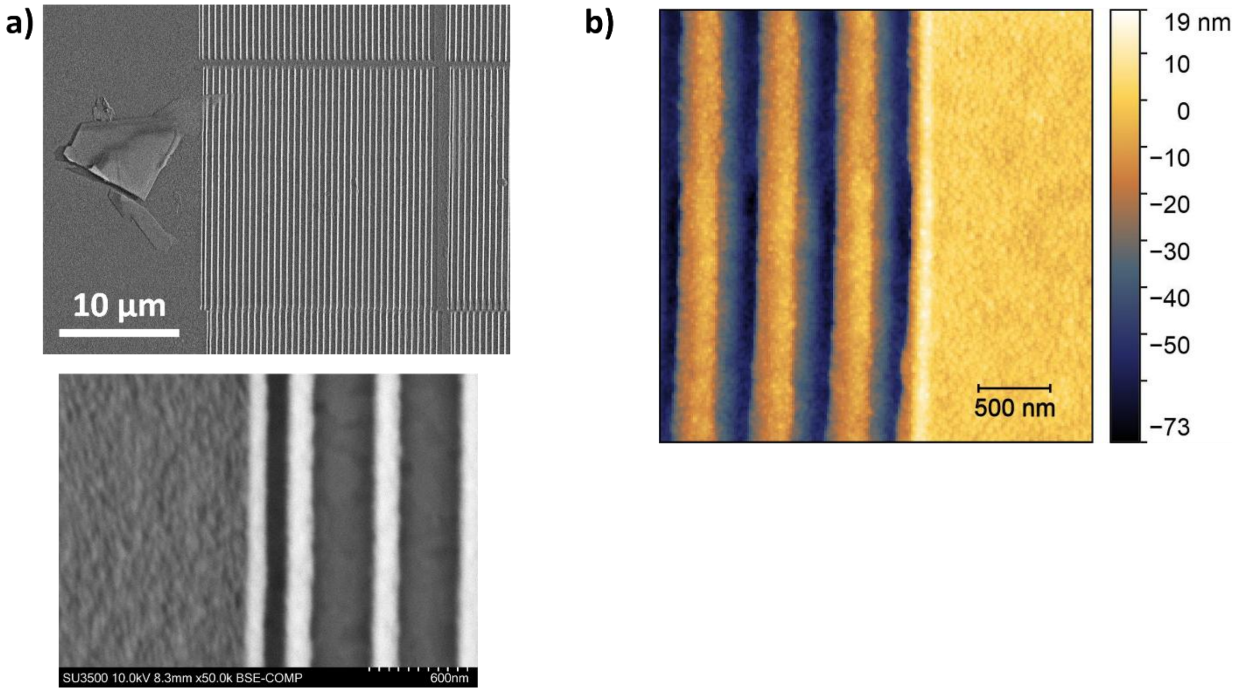

Figure S9: a) SEM images (BSE signal) acquired on the faceted nanopattern after glancing angle Au thermal evaporation. Au nanowires appear as bright high z-contrast lines, while Au deposited on flat PPA regions show lower contrast. Dark areas between Au NWs represent bare PPA regions. Inset: zoomed SEM detail at the edge of the pattern. b) Detail of a similar pattern measured by high-resolution AFM after glancing angle Au evaporation. The variation in roughness between Au on NWs and on flat PPA is due to the change in local deposition angle and thicknesses, with consequent different percolating behavior

Figure S10: Normalization of CPD profiles

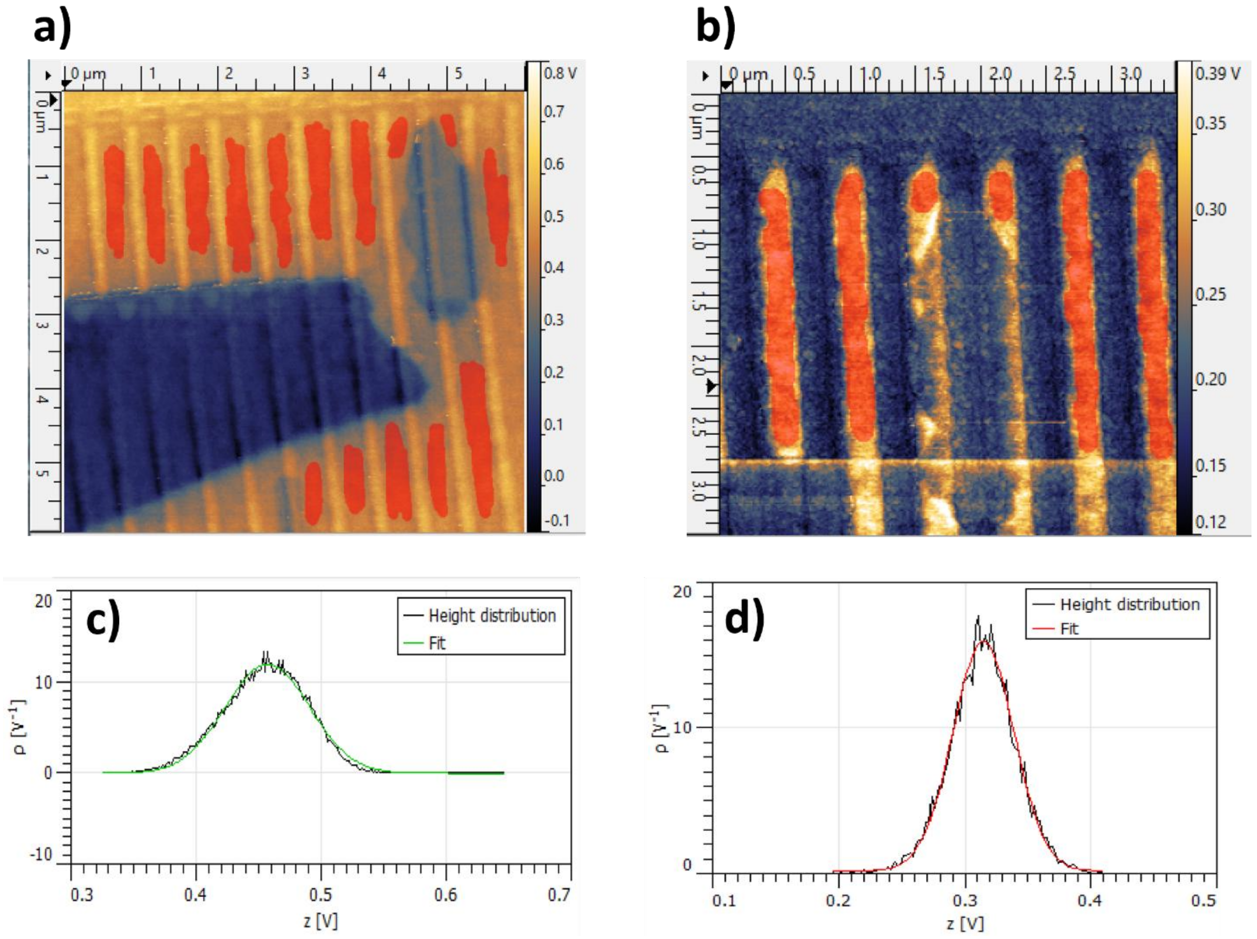

Figure S10: a-b) CPD maps reported in Figure 3b and 4b in the manuscript, with overlayed red mask. The masks here isolate the areas where bare PPA is exposed, not covered by MoS2 flakes or Gold. c-d) Histogram of CPD values extracted from the areas selected by the masking shown in a-b. Solid lines show Gaussian fits to the two histograms. Centers of the two Gaussian lie respectively at CPD_{before}=456 mV and CPD_{after}=315 mV

Figure S11: Deterministic transfer of flakes on PPA nanopatterns

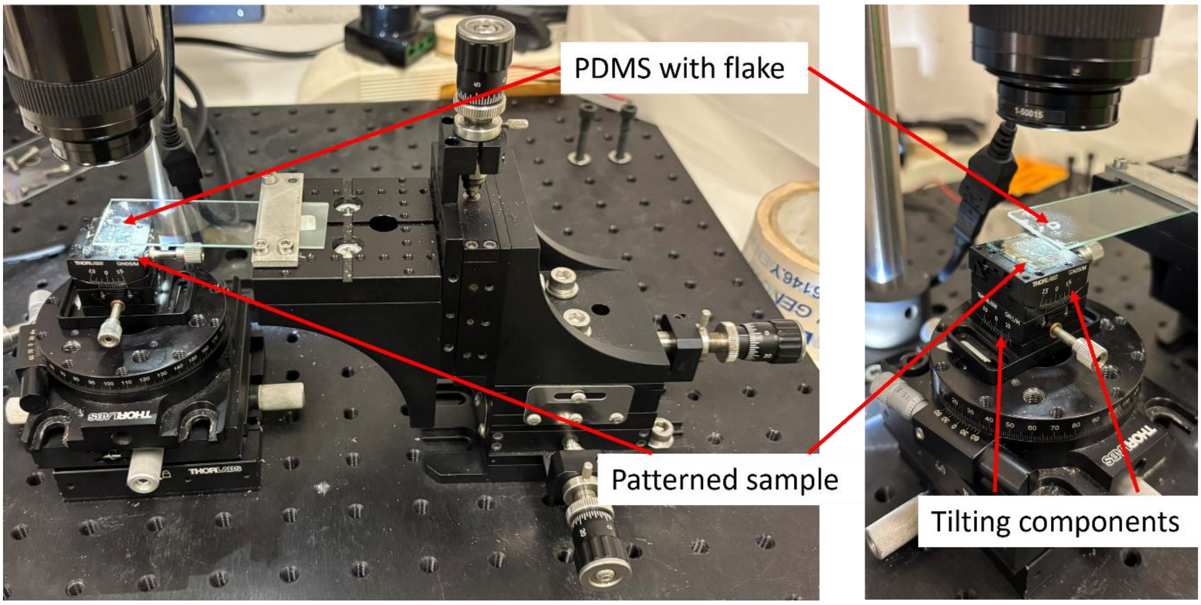

Figure S11 Picture of the custom optical microscopy setup employed for deterministic transfer of 2D layers.

The deterministic viscoelastic transfer set-up used for stamping the 2D MoS_2 layers onto the t-SPL nanopattern is presented in Figure SI 10. The patterned sample is placed onto a XY translation stage with tilting and rotating platform, while the microscope slide with the PDMS sheet is moved by a XYZ translation stage with standard micrometers. Firstly, the PDMS approaches the substrate face down and the desired flake is centered in the landing position. Then, after the contact is slowly established, finely controlling the contact wavefront, the PDMS sheet raises, releasing the flake onto the patterned sample. The entire process can be controlled by a camera connected to a microscope column[52].
